# Safety and Cost-Effectiveness of the FAM-DX Three-Dimensional CARTO Navigation System in Zero Fluoroscopy Electrophysiology Studies for Supraventricular Tachycardia: A Retrospective Cohort Study

**DOI:** 10.7759/cureus.77464

**Published:** 2025-01-15

**Authors:** Shaden Daloub, Abdusalam Elgrewi, Tala Altarawneh, Jay C Jensen, Mamdouh Y Souleymane, Khalid Abozguia

**Affiliations:** 1 Advanced Heart Failure and Heart Transplant, Kansas City University, Kansas City, USA; 2 Internal Medicine, Marshall Health, Huntington, USA; 3 Internal Medicine, Marshall University Joan C. Edwards School of Medicine, Huntington, USA; 4 Electrophysiology, Marshall University Joan C. Edwards School of Medicine, Huntington, USA

**Keywords:** carto navigation system, electrophysiology studies, fam dx, svt ablation, zero fluoroscop

## Abstract

Background

The zero fluoroscopy (ZF) ablation technique reduces radiation exposure for both medical staff and patients but typically requires specialized navigation ablation catheters. The innovative FAM-DX Three-Dimensional CARTO Navigation System enables 3D mapping without the need for these specialized catheters. This study investigates the safety, feasibility, efficacy, and cost-effectiveness of using the FAM-DX system for ZF electrophysiology (EP) studies in patients with supraventricular tachycardia (SVT).

Methods

A retrospective analysis was performed at a single center on patients who underwent EP studies using the FAM-DX system between November 2021 and December 2023. Exclusion criteria included patients under 18, pregnant women, those with recent pacemaker implantation, and individuals requiring specific ablations. Clinical data, including patient characteristics, indications, procedure details, fluoroscopy use, ablation requirements, and any procedure-related adverse events, were collected.

Results

A total of 87 consecutive patients (mean age: 53 ± 18.9 years) were included in this retrospective cohort analysis. Of these, 86 patients (98.85%) successfully underwent ZF ablation using the FAM-DX 3D navigation system, with mapping conducted in various heart regions, including the right atrium, His bundle, coronary sinus, superior vena cava, and inferior vena cava. Only one patient required fluoroscopy due to vascular access issues. Notably, in 41% of cases (36 patients), the ablation catheter was not initially needed or used during the electrophysiological study, suggesting a potential cost-saving benefit given the catheter’s cost of approximately $2,431.

Conclusions

Our study demonstrates that the FAM-DX system enables safe and efficient 3D mapping and ZF techniques for SVT EP studies. This cost-effective approach suggests that the use of ablation catheters may be unnecessary for certain patients. Further research is required to validate the broader adoption of ZF techniques and their application in more complex left-sided procedures.

## Introduction

The prevalence of supraventricular tachycardia (SVT) is 2.25 per 1,000 individuals, with females affected twice as often as males across all age groups [1]. The field of electrophysiology (EP) for treating SVT has undergone a transformative shift with the integration of 3D electro-anatomical mapping systems. The transition from traditional fluoroscopy-based catheter ablation to more advanced zero fluoroscopy (ZF) techniques is driven by several compelling factors. Conventional fluoroscopy (CF) methods involve prolonged radiation exposure, posing health risks to both patients and healthcare providers [2-5]. Additionally, the potential for long-term orthopedic injuries from the use of lead aprons among medical staff underscores the need for safer alternatives [6].

Several studies have demonstrated the safety and efficacy of minimizing or eliminating fluoroscopy during EP procedures [6,7]. While ZF EP studies using impedance-based 3D mapping systems with diagnostic catheters are feasible, magnetic-enabled systems such as CARTO (Biosense Webster, Diamond Bar, CA, USA) typically require costly Nav-enabled ablation catheters for 3D mapping. Magnetic-enabled 3D mapping provides stable and accurate results with less reliance on impedance changes compared to impedance-based systems [8]. The recent introduction of the FAM-DX module within the CARTO system allows for 3D mapping using diagnostic Nav-enabled DecaNav catheters, eliminating the need for upfront use of Nav-enabled ablation catheters.

3D mapping offers several advantages over traditional fluoroscopy-based techniques, especially in terms of efficacy and precise localization of ablation targets, such as slow pathways for atrioventricular nodal reentry tachycardia (AVNRT) ablation [4]. This ZF approach enhances safety, significantly reduces radiation exposure, and adheres to the as low as reasonably achievable (ALARA) principle for radiation dosing [5]. Furthermore, it offers substantial cost savings compared to traditional procedures, supporting its broader adoption in clinical practice [9].

Magnetic-enabled 3D mapping systems have transformed the management of SVT in medical centers worldwide [10]. This study aims to assess the efficacy and safety of using the CARTO PRIME V7 module FAM-DX in EP studies and ablations for SVT. The study will evaluate the potential of this innovative technology to reduce radiation exposure for both patients and medical staff, eliminate orthopedic risks from lead apron use, and optimize cost efficiency through selective use of costly Nav-enabled ablation catheters.

## Materials and methods

Study design and patient population

This was a single-center, retrospective cohort study conducted at Marshall University Joan C. Edwards School of Medicine, Huntington, USA. Following IRB approval, we enrolled patients aged 18 years and older who underwent EP studies for SVT using the FAM-DX 3D CARTO Navigation system between November 2021 and December 2023. Inclusion criteria were as follows: patients undergoing an EP study for SVT, aged 18 years or older, and from all ethnic groups. Exclusion criteria included individuals under 18 years, pregnant patients, and those requiring procedures that necessitated the upfront use of an ablation catheter (e.g., atrioventricular node ablation, atrial flutter, atrial fibrillation, premature ventricular contractions, or ventricular tachycardia ablations). Patients with pacemakers implanted within the last six months were also excluded to prevent the risk of lead dislodgment and displacement. Prior to the procedure, each patient underwent a uniform evaluation, including a detailed history, clinical symptom assessment, standard blood tests, electrocardiography, and comprehensive echocardiography. All anti-arrhythmic medications were discontinued at least five half-lives before the procedure.

Data collection

Data were collected from various sources, including electronic medical records (Sorian, North Kansas City, MO, USA), the EP recording system (WorkMate Claris Electrophysiology System, Abbott, Plymouth, MN, USA), and the physiological tracings recorder (Mac-Lab™ Hemodynamic Recording System, GE Healthcare, Chicago, IL, USA). This data included patient demographics (age and gender), medical history, arrhythmia documentation, hospitalization details, and medication use. Additionally, the data covered procedure-related information such as date and time, vascular access, EP study findings, procedure duration, use of 3D mapping, radiation exposure (including fluoroscopy time and dose), need for ablation, and the procedural outcome, including success and complications. Descriptive statistics were used to summarize patient demographics and clinical outcomes following data collection.

Procedure details

All procedures were performed by a single electrophysiologist, with support from a training fellow. The analysis included all procedures, including those conducted during the initial phase of the ZF approach. Each EP study using the FAM-DX system was documented step by step, beginning with patient preparation, followed by mapping and navigation during the procedure, and concluding with post-procedural care.

Patients were prepped and draped in the standard sterile manner. Vascular access was obtained in the right femoral vein using two 8-French and one 7-French sheath, with ultrasound guidance and the Seldinger technique to minimize the risk of vascular access-related complications [11]. A DecaNav catheter was employed to create a 3D CARTO mapping geometry of the right atrium, including the His bundle, coronary sinus (CS), inferior vena cava (IVC), and superior vena cava (SVC). Catheters were placed in various locations within the heart. Pacing was performed at twice the diastolic threshold. Atrial and ventricular burst pacing, as well as programmed electrical stimulation with up to two extra stimuli, were performed in baseline, isoproterenol (up to 5 mcg/min), and recovery states.

If the EP study was positive for tachycardia, an ablation catheter was used to perform ablation at the operator’s discretion. After ablation, pacing was repeated in both the baseline and isoproterenol (5 mcg/min) states. The success of the procedure was defined as the inability to reinduce tachyarrhythmia or the elimination of the tachycardia substrate.

Follow-up

Upon returning to the ward, patients were promptly assessed with a detailed physical examination, vital signs monitoring, and ECG monitoring. Routine follow-up visits were scheduled at the EP clinic according to the local protocol.

## Results

**Figure 1:**
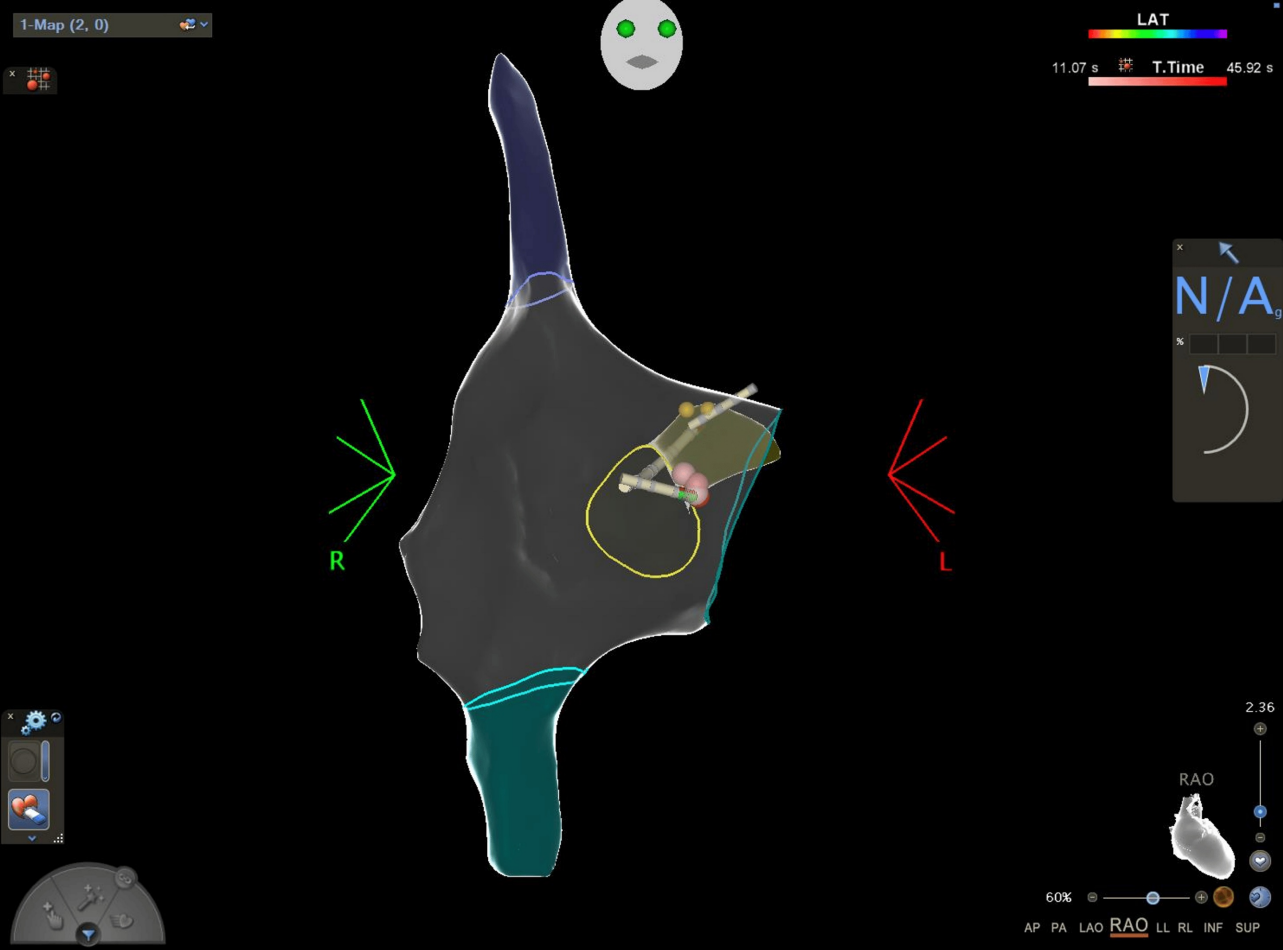
Right anterior oblique This image shows a 3D map of the right atrium, including the SVC (purple), IVC (green), and CS (yellow structure). Red dots represent ablation points at the slow pathway, while yellow spots indicate the His bundle. The CRD 2 is positioned at the His bundle, and the DecaNav catheter is placed in the CS. CRD, catheter radiofrequency device; CS, coronary sinus; IVC, inferior vena cava; SVC, superior vena cava

**Figure 2:**
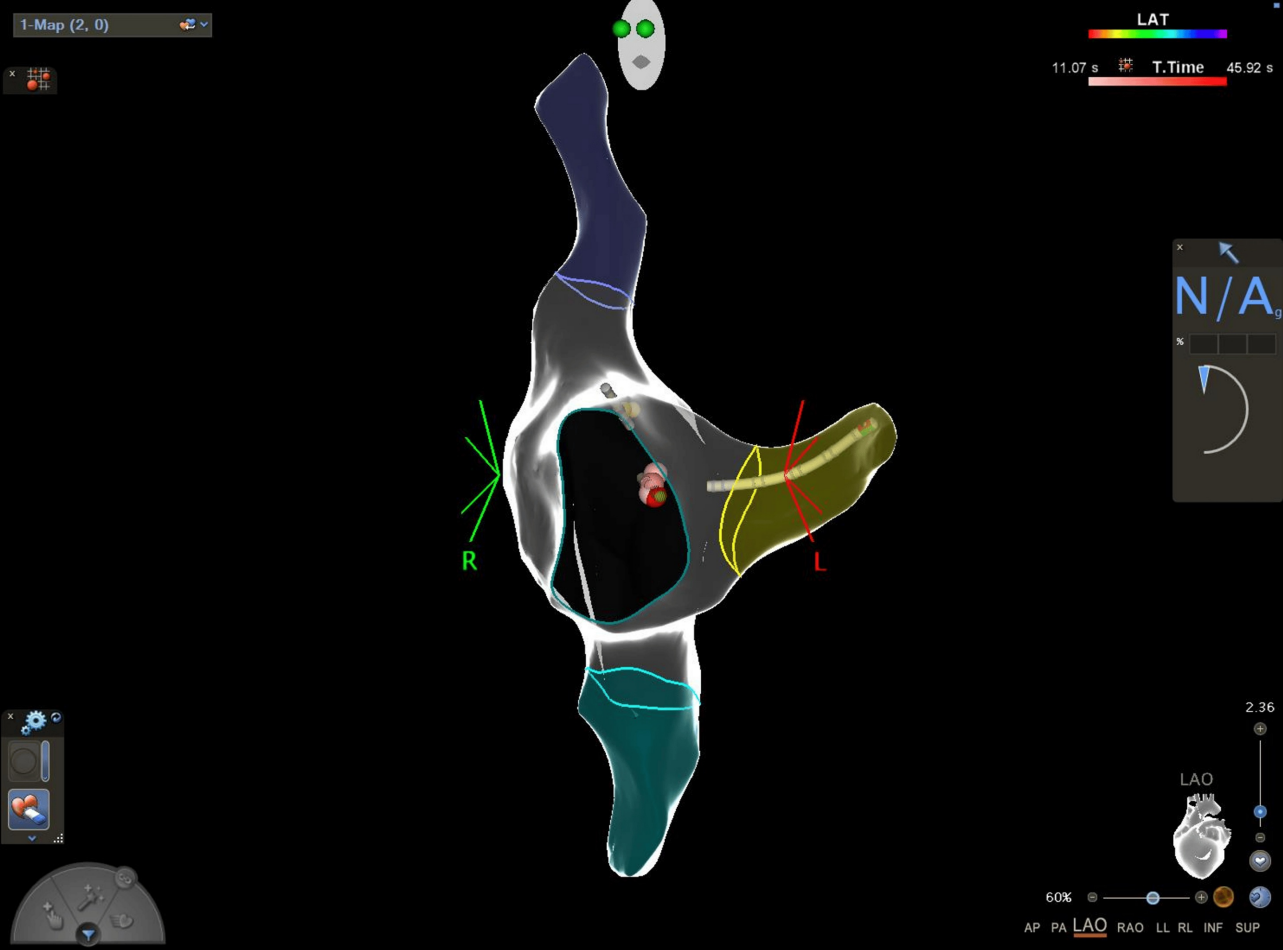
Left anterior oblique This image displays a 3D map of the right atrium, including the SVC (purple), IVC (green), and CS (yellow structure). Red dots represent ablation points at the slow pathway, while yellow spots denote the His bundle. The CRD 2 is positioned at the His bundle, and the DecaNav catheter is placed in the CS. CRD, catheter radiofrequency device; CS, coronary sinus; IVC, inferior vena cava; SVC, superior vena cava

A total of 87 patients were included in the study, with a mean age of 53 ± 18.9 years. Males comprised 36% and females 64% of the study population. All participants were identified as white. The average BMI was 30.9 ± 6.8 kg/m², indicating a tendency toward overweight status within the cohort (Table 1). A major focus of the study was the use of ZF ablation with the FAM-DX 3-D navigation system, which was employed in 98.85% of cases (Figure 1 shows the 3D mapping of the right atrium, His region, CS, SVC, and IVC; Figure 1 represents the right anterior oblique view, and Figure 2 represents the left anterior oblique view). Only one patient (1.15%) required fluoroscopy to facilitate vascular access due to complex venous anatomy.

**Table 1:** Baseline characteristics CAD, coronary artery disease; COP, chronic obstructive pulmonary disease; DM, diabetes mellitus; ESKD, end-stage kidney disease; HTN, hypertension; LVEF, left ventricular ejection fraction; OSA, obstructive sleep apnea

Overall population	Average (N = 87)
Age	53 ± 18.9
Race	White (100%)
Male	31 (36%)
Female	56 (64%)
HTN	40 (46%)
Hyperthyroidism	1 (1%)
Hypothyroidism	11 (13%)
DM	16 (18%)
CAD	14 (16%)
COPD	6 (11%)
OSA	4 (5%)
ESKD	0%
LVEF on echo	56.32 +/- 9.94
Medication	
Beta-blocker	58 (66%)
Calcium channel blocker	14 (16%)
Amiodarone	1 (1%)
Flecainide	12 (14%)

**Figure 3:**
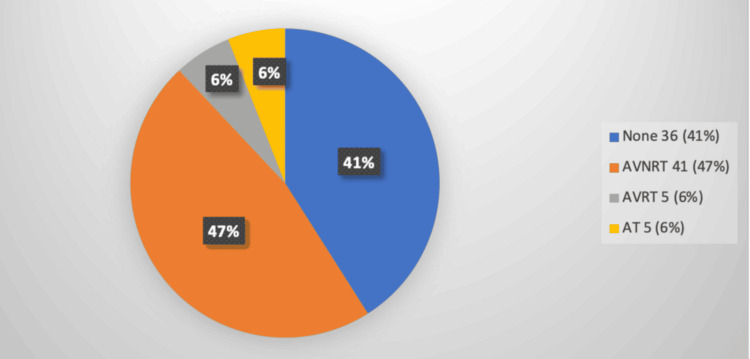
Types of arrhythmias AT, atrial tachycardia; AVNRT, atrioventricular nodal reentry tachycardia; AVRT, atrioventricular reentrant tachycardia

Regarding arrhythmias, AVNRT was the most common, observed in 41 patients (47%), followed by atrial tachycardia in five patients (5.75%) and atrioventricular re-entrant tachycardia in five patients (5.75%). Notably, 36 patients (41%) were found to have no arrhythmia (Figure 3), and therefore, no ablation catheter was used in this subgroup, highlighting the cost-saving advantage of the FAM-DX 3-D navigation system. Radiofrequency ablation was the treatment modality for all patients (100%), reflecting a clear preference for this technique over cryoablation. The study recorded a 100% procedural success rate, with no significant acute complications, underscoring the safety and efficacy of the procedures performed. 

## Discussion

CF has long been employed in ablation therapy for SVT, offering both advantages and disadvantages. CF provides real-time visualization, aiding in the guidance of procedures, accurate catheter placement, and successful outcomes. However, it also presents significant risks, primarily from radiation exposure, which affects not only patients but also medical staff.

Medical professionals, especially physicians and cardiologists, are at increased risk of developing various types of cancer due to their exposure to radiation during cardiac procedures. Studies have indicated that repeated exposure to X-rays in the medical field is linked to elevated risks of leukemia, skin cancer, female breast cancer, and potentially thyroid cancer [12]. For example, a meta-analysis found a clear association between occupational radiation exposure and increased risks of leukemia among radiologic technologists [13]. Likewise, a study by Rajaraman et al. reported elevated risks of skin cancer in physicians with long-term radiation exposure [14]. Evidence also suggests a link between radiation exposure and an increased risk of breast cancer in female physicians and cardiologists, as demonstrated by Ronckers et al. [15]. Furthermore, Pearce et al. highlighted potential thyroid cancer risks associated with radiation exposure among medical professionals [16]. These findings emphasize the need for effective radiation protection measures in healthcare settings to minimize occupational hazards and safeguard medical personnel’s health.

In addition to the occupational hazards linked to radiation, interventional cardiologists and electrophysiologists are also prone to orthopedic complications due to the heavy lead protective equipment worn during procedures. This protective gear often leads to musculoskeletal issues such as back pain and spinal complaints [17]. Despite efforts to mitigate these concerns by reducing fluoroscopy doses and limiting cine imaging, CF continues to pose potential health risks. As a result, there is growing interest in exploring alternative techniques that eliminate fluoroscopy.

One promising approach involves integrating 3D electro-anatomical mapping and ultrasound imaging to achieve ZF procedures. By relying on these advanced technologies, clinicians can navigate cardiac anatomy and guide interventions without radiation exposure. This not only minimizes the risk of radiation-related health complications but also alleviates the orthopedic issues associated with heavy lead shielding [6].

Several studies have demonstrated the efficacy and safety of ZF techniques in cardiac interventions. For instance, a survey by Haegeli et al. [18] confirmed the feasibility and effectiveness of ZF ablation procedures using 3D mapping systems. Similarly, a systematic review by Kawakami et al. [19] concluded that ZF approaches significantly reduced radiation exposure without compromising procedural success rates.

The introduction of 3D electro-anatomical mapping has revolutionized cardiac EP, offering precise guidance and mapping capabilities during procedures. This technology includes two types of mapping systems: impedance-based and magnetic-based, each with its unique characteristics [20].

The impedance-based approach is valued for its cost-effectiveness, as it does not require expensive navigation-enabled ablation equipment or advanced mapping catheters. However, its stability is impacted by the dynamic nature of cardiac structures, which causes fluctuations in impedance. This necessitates the use of a stable reference catheter within the heart.

On the other hand, the magnetic-based approach requires a substantial initial investment in navigation-enabled ablation systems and advanced mapping catheters, which can increase procedural costs. However, it provides unparalleled accuracy and stability, enhancing procedural precision compared to impedance-based methods [21].

Both approaches offer distinct advantages and limitations. Their integration into ZF strategies represents a significant advancement in SVT ablation therapy, allowing clinicians to reduce reliance on fluoroscopy and avoid radiation exposure risks for both patients and operators.

Research supports the efficacy and safety of these mapping technologies in guiding ablation procedures. A meta-analysis by Turagam et al. [22] found that magnetic-based mapping systems significantly improve procedural success rates and reduce fluoroscopy time compared to impedance-based approaches.

As advancements continue to refine these mapping technologies, their integration into clinical practice holds promise for optimizing SVT ablation outcomes while prioritizing patient and operator safety.

FAM-DX, a recent innovation introduced by Biosense Webster, represents a groundbreaking advancement in cardiac EP, enabling fast anatomical 3D mapping using a diagnostic catheter, such as the new DecaNav diagnostic catheter. This technology eliminates the traditional need for expensive upfront ablation catheters or sophisticated mapping catheters. By utilizing a magnetic-enabled system, FAM-DX offers high-resolution 3D mapping for EP studies, even in cases where the requirement for an ablation catheter is uncertain.

The utilization of FAM-DX provides several distinct advantages over conventional impedance-based 3D mapping methods. First, it offers more accurate and stable 3D mapping, improving procedural precision and efficacy. Additionally, the adoption of FAM-DX could lead to significant cost savings, particularly in situations where ablation is not necessary.

We believe that ZF techniques offer significant advantages over fluoroscopy in all types of procedures due to their enhanced safety profile. Fluoroscopy may still be necessary in cases of patients with recently implanted pacemakers, where there is a risk of lead displacement, or in cases requiring epicardial access, such as epicardial ablation for ventricular tachycardia.

Our study is the first to apply this standard approach to all EP studies related to SVT, regardless of the need for ablation. Our findings support the safety and feasibility of using FAM-DX with the DecaNav diagnostic catheter in this context. Notably, 41% of participants in our study did not require ablation, highlighting the cost-effectiveness of this approach and offering potential savings given the catheter’s cost of approximately $2,431. Furthermore, only one patient required minimal fluoroscopy due to tortuous vascular access.

Our data clearly demonstrate that FAM-DX provides a feasible, safe, and cost-effective approach to performing ZF EP studies using magnetic-enabled 3D mapping. However, it is important to acknowledge several limitations in our research, including the lack of double-blinding and the need for a larger sample size. Additionally, the learning curve and time required to master this technique, especially for novice electrophysiologists, remain unknown.

## Conclusions

Our study demonstrated the efficacy of the FAM-DX magnetic navigation system in facilitating the adoption of magnetic-enabled 3D mapping and ZF techniques during EP studies targeting SVT. By seamlessly integrating these advanced technologies, the FAM-DX system offers a safe and efficient approach to guiding procedures while minimizing radiation exposure. This not only enhances patient safety but also streamlines workflow for clinicians, ultimately optimizing procedural outcomes through magnetic-enabled 3D mapping, as opposed to impedance-based 3D mapping. Furthermore, our findings highlight the cost-effectiveness of using the FAM-DX navigation system in ZF procedures. By eliminating the need for an initial ablation catheter, especially in patients who do not require ablation, substantial cost savings can be achieved. This demonstrates the potential of the FAM-DX system to reduce healthcare costs associated with unnecessary instrument utilization, offering a prudent economic strategy for healthcare providers and institutions.

It is important to note the limitations of our study, including its single-center, non-randomized design, the lack of double-blinding, and the need for a larger sample size. Large, randomized studies are necessary to further validate these findings.
